# From Rescue Therapy to Mechanism-Based Sequencing in Refractory Myasthenia Gravis: A Narrative Review with an Illustrative Clinical Case

**DOI:** 10.3390/jcm15197369

**Published:** 2026-09-22

**Authors:** Aleksandr Ilves, Vera Nikitin

**Affiliations:** Department of Neurology, Sheba Medical Center, Ramat Gan 52621, Israel; ailves@hotmail.com

**Keywords:** myasthenia gravis, refractory myasthenia gravis, acetylcholine receptor antibodies, treatment sequencing, FcRn inhibition, complement inhibition, B-cell depletion, nipocalimab

## Abstract

Refractory acetylcholine receptor antibody-positive generalized myasthenia gravis (AChR-Ab-positive gMG) is clinically heterogeneous, and evidence on how to select a therapeutic mechanism after incomplete response to a previous targeted class remains limited. This narrative review synthesizes guidelines, randomized trials, extension studies, meta-analyses, observational evidence, and emerging translational data identified through targeted searches updated to 21 June 2026, with a final bibliographic check through 10 August 2026. We examine conventional immunosuppression, rescue therapy, B-cell and B-lineage depletion, terminal complement inhibition, and neonatal Fc receptor (FcRn) inhibition as interventions acting at different levels of the pathogenic cascade. We propose a framework in which prior response, nonresponse, intolerance, corticosteroid dependence, respiratory vulnerability, and rescue-therapy dependence are interpreted as mechanistic clues rather than as a fixed treatment ladder. An illustrative case of severe refractory AChR-Ab-positive gMG showed incomplete control after conventional immunosuppression, rituximab, thymectomy, and terminal complement inhibition, with reproducible but transient benefit from plasma exchange. This pattern supported a mechanistic rationale for FcRn inhibition, although overlapping treatments and single-case design preclude causal attribution. Mechanism-based sequencing should be viewed as structured decision support rather than a validated algorithm. Prospective switching studies and predictive biomarkers are needed to optimize individualized treatment sequencing.

## 1. Introduction

Myasthenia gravis (MG) is an autoimmune disorder of the neuromuscular junction (NMJ) characterized by fluctuating fatigable weakness. In generalized MG (gMG), weakness may involve ocular, bulbar, limb, axial, and respiratory muscles. The most common immunological subtype is acetylcholine receptor antibody-positive (AChR-Ab-positive) gMG, in which pathogenic immunoglobulin G (IgG) antibodies impair neuromuscular transmission through receptor blockade, antigenic modulation with accelerated acetylcholine receptor (AChR) loss, and complement-mediated postsynaptic injury [1,2]. Current guidance emphasizes individualized treatment according to antibody status, disease severity, comorbidities, prior response, safety, and treatment goals [3,4].

Long-term management usually combines symptomatic therapy, corticosteroids (CS), conventional immunosuppressive (IS) therapy, short-term immunomodulation with intravenous immunoglobulin (IVIG) or plasma exchange (PLEX), thymectomy in appropriate patients, and targeted immunotherapy when escalation is required [3,4]. In refractory disease, however, the problem is not simply persistent weakness. It is the failure to achieve durable control without excessive treatment burden, including CS dependence, intolerance of steroid-sparing agents, recurrent exacerbations or crisis, respiratory vulnerability, or repeated IVIG/PLEX use [5,6,7].

The therapeutic landscape has expanded with agents acting at distinct levels of the pathogenic cascade. Terminal complement inhibition, FcRn-mediated IgG reduction, and B-cell or B-lineage targeting have each been supported by randomized trials or contemporary evidence syntheses in gMG [8,9,10,11,12,13,14,15,16,17,18]. These advances create a practical question that remains largely unanswered: once a response to one mechanism is incomplete, transient, or intolerable, how should the next mechanism be chosen?

The premise of this review is that refractory AChR-Ab-positive gMG should not be approached only as a therapeutic ladder. Several pathogenic layers may remain active to different degrees, including autoantibody generation, circulating IgG persistence, antibody binding at the NMJ, complement-mediated injury, and treatment-limiting toxicity. A partial or absent response to one intervention may therefore indicate not abandonment of the antibody-mediated model, but a layer that has not yet been adequately controlled.

We therefore adopt a different point of observation. Rather than asking only which drug should follow the previous one, we ask what the longitudinal treatment trajectory reveals about unresolved immunopathology. Prior benefit, nonresponse, intolerance, CS dependence, respiratory vulnerability, and rescue-therapy dependence are treated as clinical clues. The review defines refractory MG as a heterogeneous state, relates pathogenic layers to therapeutic targets, examines major therapeutic mechanisms through their interpretive value, proposes a mechanism-based sequencing framework, and illustrates the approach with a severe refractory case.

## 2. Literature Search and Source Selection

This article was designed as a narrative review because its primary objective was not to estimate a pooled treatment effect or to compare predefined interventions systematically, but to integrate heterogeneous evidence relevant to a clinical question for which prospective sequencing trials are largely unavailable: how prior treatment response, nonresponse, and intolerance may inform selection of a subsequent therapeutic mechanism in refractory AChR-Ab-positive generalized myasthenia gravis. A narrative approach allowed consideration of randomized trials, extension studies, guidelines, observational evidence, mechanistic studies, and emerging translational data within a common clinical framework. We recognize that this approach is inherently more susceptible to selection bias than a systematic review and does not provide the reproducibility or formal evidence grading of a prespecified systematic-search protocol.

Targeted literature searches were performed in PubMed/MEDLINE, the Cochrane Library, and ClinicalTrials.gov and were supplemented by official guideline and regulatory sources. Searches were last updated on 21 June 2026, with a final targeted bibliographic check through 10 August 2026. PubMed/MEDLINE was used as the principal biomedical database because the review focused primarily on clinical, therapeutic, and translational literature in myasthenia gravis; the Cochrane Library was used to identify systematic evidence syntheses, while ClinicalTrials.gov and regulatory sources were used particularly to verify the status of recent or emerging therapeutic approaches. Web of Science and Scopus were not included because the review was designed as a targeted narrative synthesis rather than an exhaustive multidisciplinary citation search. Their omission is acknowledged as a potential source of incomplete retrieval.

Search concepts combined myasthenia gravis or generalized myasthenia gravis with terms related to refractory or highly active disease, treatment sequencing or switching, corticosteroids, conventional immunosuppression, intravenous immunoglobulin, plasma exchange, B-cell or B-lineage therapy, complement inhibition, FcRn inhibition, biomarkers, and investigational antigen-specific or cellular therapies. Search terms were adapted to the terminology and syntax of the individual sources. Priority was given to contemporary clinical guidelines, pivotal randomized controlled trials, extension studies, systematic reviews and meta-analyses, and peer-reviewed observational studies. Mechanistic and translational studies were included when they directly informed interpretation of therapeutic targets or treatment-response patterns. Registry records and conference-level reports were considered only for emerging therapies when peer-reviewed data were unavailable and were identified as such.

Sources were selected when they directly informed at least one of the following domains: definition and clinical burden of refractory gMG; efficacy or safety of relevant therapeutic classes; biological mechanisms relevant to treatment response; evidence regarding switching, sequencing, or combination therapy; or interpretation of the illustrative clinical case. Publications that were not directly relevant to these questions, duplicated more complete reports, or had been superseded by subsequent peer-reviewed publications were not preferentially retained. Because this was a narrative rather than a systematic review, duplicate independent screening, formal study-level risk-of-bias assessment, and quantitative evidence synthesis were not performed. Throughout the review, we distinguish conclusions supported directly by clinical trial or observational evidence from mechanistic inferences and clinical interpretations regarding therapeutic sequencing.

## 3. Refractory AChR-Ab-Positive gMG as a Heterogeneous Clinical State

Refractory myasthenia gravis lacks a universally accepted definition, and this absence of a single standard has practical consequences. Tran et al. compared five published refractory MG criteria and showed that different definitions identify different proportions of patients and differ in their agreement and association with disease severity, fatigue, and quality-of-life measures [7]. Across definitions, refractoriness is usually captured by overlapping features: persistent symptoms despite adequate immunotherapy, relapse during CS tapering, treatment intolerance, recurrent exacerbations or crisis, chronic need for IVIG or PLEX, and continued functional impairment despite apparently appropriate treatment [5,6,7].

The estimated frequency of refractory MG varies because definitions differ. Suh, Goldstein, and Nowak classified 19 of 128 patients in a tertiary neuromuscular clinic cohort as refractory, corresponding to 14.8% [5]. Cortes-Vicente et al., using data from the Spanish MG Registry, reported that 8.5% of patients were drug-refractory [19]. Rath et al. and Jeong et al. also showed that treatment-refractory patients have greater clinical burden, including more frequent exacerbations, need for rescue treatment, hospitalization, crisis, and infection-related complications [20,21]. Together, these studies support the view that refractory MG affects a minority of patients but represents a clinically distinct high-burden subgroup.

A purely symptom-based definition is insufficient. Persistent weakness, ocular symptoms, bulbar involvement, respiratory vulnerability, and impaired daily functioning are central, but refractory MG is also defined by treatment context. A patient who cannot taper prednisone without worsening, cannot tolerate conventional CS-sparing agents, or requires repeated IVIG or PLEX may be refractory even if some symptoms appear partially controlled [5,6,7]. The Israeli national guideline explicitly includes the inability to reduce CS to 10 mg prednisone equivalent or less because of continued weakness or exacerbations requiring PLEX or IVIG within its refractory/high disease activity framework [4].

The distinction between active, highly active, and refractory MG is increasingly relevant in the era of targeted therapy. German and Israeli national guidelines frame highly active or refractory gMG as a severe disease state requiring escalation beyond symptomatic treatment, with decisions shaped by antibody status, disease activity, prior medication history, and the need for rapid-onset therapy in severe exacerbation or crisis [4,22]. International consensus guidance similarly treats myasthenic crisis and severe exacerbation as indications for urgent immunomodulatory therapy such as IVIG or PLEX [3].

Refractory MG should therefore be viewed as a heterogeneous clinical state rather than a single biological category. Some patients may be refractory because upstream autoantibody production remains insufficiently controlled; others because circulating IgG persists despite cellular immunotherapy; others because downstream complement-mediated injury remains active; and others because treatment toxicity prevents adequate therapy. This is not a formal biomarker-based classification, but it follows from the therapeutic targets in AChR-Ab-positive MG and from the heterogeneous clinical definitions used in published cohorts and guideline frameworks.

For the purposes of this review, refractory AChR-Ab-positive gMG refers to gMG with persistent clinically meaningful disease activity, treatment intolerance, CS dependence, recurrent exacerbations or crisis, ongoing IVIG/PLEX requirement, or incomplete control despite conventional and/or targeted immunotherapy. This broad working definition is intentionally clinical rather than strictly trial-based, because our central question is how prior treatment response should guide selection of the next therapeutic mechanism.

## 4. Pathogenic Layers and Therapeutic Targets in AChR-Ab-Positive gMG

AChR-Ab-positive gMG can be viewed as a layered IgG-mediated autoimmune disease in which related but separable processes—antibody generation, circulating IgG persistence, antibody binding, complement activation, and postsynaptic injury—can be targeted at different points. In refractory disease, this layered structure matters: incomplete response to one mechanism does not necessarily mean that the disease has ceased to be antibody-mediated. It may instead suggest that another process within the cascade remains active. Passive-transfer and immunopathological studies established early that AChR antibodies can reproduce myasthenic weakness and damage the postsynaptic membrane, supporting the central role of humoral autoimmunity in AChR-Ab-positive disease [1,2,23].

The core immunopathological event is production of pathogenic IgG autoantibodies directed against the AChR at the postsynaptic membrane of the NMJ. These antibodies impair neuromuscular transmission through direct functional interference with receptor activity, cross-linking and accelerated internalization of AChRs, and complement-mediated injury of the postsynaptic membrane [1,2,23]. The final common pathway is reduction in the safety factor for neuromuscular transmission. Loss of functional AChRs reduces the amplitude of endplate potentials, while structural postsynaptic injury further compromises transmission efficiency, explaining the characteristic fatigability and fluctuation of weakness [1,23].

This cascade creates several therapeutically relevant targets. Conventional IS therapies broadly suppress immune activation and may reduce autoantibody generation over time, but their onset is delayed, and their effects may be incomplete in refractory disease [3,4,24]. B-cell-directed therapy targets cellular sources or precursors of autoantibody generation, but such therapy does not directly eliminate mature plasma cells and does not rapidly remove IgG already present in the circulation. Rescue therapies intervene at the humoral level: PLEX can rapidly remove circulating pathogenic antibodies and other plasma factors, while IVIG provides short-term immunomodulation during exacerbations, crises, perioperative stabilization, or bridging to slower therapies [3,4].

Complement inhibition targets a downstream effector mechanism rather than autoantibody production or the circulating antibody pool itself. In AChR-Ab-positive MG, complement activation contributes directly to postsynaptic membrane injury, providing the biological rationale for terminal complement component 5 (C5) inhibition with eculizumab, ravulizumab, and zilucoplan [2,8,9,10]. However, complement blockade does not remove circulating pathogenic IgG, prevent antibody binding to AChR, or suppress the B-cell and plasma-cell compartments that generate autoantibodies.

FcRn inhibition intervenes at a different humoral level. FcRn protects IgG from lysosomal degradation and prolongs IgG half-life; pharmacological FcRn blockade accelerates IgG catabolism and lowers total IgG, including pathogenic AChR antibodies [11,12,13,14,25,26]. Mechanistically, FcRn inhibition differs from complement inhibition because it reduces the circulating IgG burden that can feed several antibody-mediated pathogenic mechanisms simultaneously.

Thus, treatment failure in refractory AChR-Ab-positive gMG should not be read only as failure of a drug. It can also indicate where the cascade remains active: autoantibody production, circulating IgG persistence, antibody binding at the NMJ, complement-mediated injury, impaired neuromuscular transmission, treatment toxicity, or a combination of these factors. This interpretive step is the basis for mechanism-based sequencing.

## 5. Conventional Immunosuppression in gMG: Mechanistic Role and Persistent Limitations

Conventional IS remains part of the long-term foundation of gMG care, but in a sequencing framework it represents broad upstream immune modulation rather than rapid rescue or precise effector blockade. CS often improve symptoms, and nonsteroidal IS agents are used to reduce cumulative CS exposure. Treatment choice is shaped by disease severity, comorbidities, adverse-effect profile, expected time to benefit, reproductive considerations, and clinical experience [3,4].

Mechanistically, these therapies may reduce autoreactive immune activation and autoantibody generation over time, but they do not rapidly remove circulating pathogenic IgG, eliminate established long-lived plasma cells, or block complement-mediated postsynaptic injury. This distinction explains why conventional IS may remain necessary background treatment while being insufficient as the sole strategy in highly active or respiratory-vulnerable disease.

The conventional evidence base is most useful here because it defines these therapies as background disease-control strategies. AZA has the strongest historical steroid-sparing evidence, MMF is widely used despite less supportive short-term randomized trials, MTX and calcineurin inhibitors have narrower or more variable data, and CS tapering requires structured monitoring because relapse during reduction is common [24,27,28,29,30,31,32,33,34,35,36,37]. These differences matter clinically, but they do not by themselves solve the sequencing problem after incomplete response to a targeted mechanism.

For the present framework, the key question is whether adequate exposure has produced durable and tolerable control. Persistent CS dependence, relapse during tapering, intolerance of steroid-sparing therapy, recurrent rescue-therapy need, or continuing bulbar or respiratory symptoms should prompt reassessment of which pathogenic layer remains active and whether a more targeted mechanism is required.

## 6. Rescue Therapy with IVIG and PLEX: Rapid Control, Bridging, and a Marker of Refractory Disease

IVIG and PLEX are established short-term immunomodulatory therapies in MG, especially during exacerbations, impending or manifest myasthenic crisis, perioperative stabilization, and bridging while slower IS therapies take effect [3,4]. They differ from conventional CS-sparing IS because they can be used when bulbar, respiratory, or rapidly progressive generalized weakness requires immediate intervention. In refractory MG, however, acute stabilization and long-term disease control are related but distinct problems; success in one domain should not be mistaken for success in the other.

PLEX rapidly removes circulating antibodies and other plasma factors. Its early use in acquired MG supported the functional relevance of circulating anti-AChR antibodies, and later clinical studies established PLEX as an effective short-term intervention in worsening MG [38,39]. IVIG acts less as antibody removal and more as broad immunomodulation, affecting Fc receptor signaling, complement activity, cytokine networks, immune-cell function, and pathogenic autoantibody effects [40].

Comparative evidence supports clinical benefit for both IVIG and PLEX in acute or worsening MG, while leaving room for individualized choice. Randomized studies, systematic reviews, and more recent syntheses differ in whether they favor PLEX in selected acute or peri-thymectomy settings or emphasize uncertainty about uniform superiority [39,41,42,43]. In practice, the decision is shaped by urgency, vascular access, hemodynamic stability, renal function, thrombotic and infection risk, availability, and local expertise. Maintenance IVIG has a more mixed evidence base, with trial results varying by design, comparator, treatment setting, and outcome [44,45,46].

In a sequencing framework, repeated IVIG or PLEX use has clinical and mechanistic meaning. Clinically, it marks insufficient durable control when used repeatedly to prevent deterioration or bridge between relapses. Mechanistically, reproducible but transient improvement after PLEX is especially informative because it suggests that circulating humoral mediators remain clinically relevant. It does not prove that IgG is the only driver, because PLEX also removes complement components and other plasma factors.

Repeated dependence on rescue or bridge therapies should therefore trigger a search for durable control rather than be accepted as a satisfactory endpoint. A recurrent short-term response to humoral rescue may support consideration of an IgG-burden reduction strategy such as FcRn inhibition, while preserving the distinct role of IVIG and PLEX for acute stabilization.

## 7. B-Cell and B-LINEAGE Depletion: Upstream Control and Mechanisms of Incomplete Response

B-cell and B-lineage depletion are rational strategies in autoimmune MG because humoral immune responses are central to disease biology. B cells serve as precursors of antibody-secreting cells and also shape autoreactive immunity through antigen presentation, cytokine production, and T-cell interaction [47,48]. Their clinical effect varies by antibody subtype, disease duration, immune compartment, and depth of targeting [48]. In refractory AChR-Ab-positive gMG, partial or absent response should therefore be interpreted cautiously: it may reflect incomplete control of the relevant antibody-producing compartment rather than failure of the antibody-mediated disease model.

RTX depletes CD20-positive naive and memory B cells, but mature plasma cells generally lack CD20 and are not directly eliminated by anti-CD20 therapy [49,50]. This provides a mechanistic explanation for delayed, partial, or absent benefit: RTX may reduce future autoreactive B-cell responses without rapidly eliminating established autoantibody production or the circulating pathogenic IgG pool. Long-lived plasma cells can continue antibody production despite successful peripheral B-cell depletion [50].

Clinical data also depend on disease subtype and timing. The most consistent RTX benefit has been reported in MuSK-Ab-positive MG, commonly mediated by IgG4 antibodies, and these data should not be extrapolated mechanically to AChR-Ab-positive disease [51,52]. AChR-Ab-positive gMG is usually mediated by complement-fixing IgG1 and IgG3 antibodies and often involves thymic immune pathology and complement-mediated injury [2,23]. In this context, RTX responses are more heterogeneous and may depend on disease stage, immune architecture, and the antibody-producing compartment [53,54,55].

The randomized evidence illustrates this heterogeneity quantitatively rather than resolving it. In BeatMG, conducted in mild-to-moderate AChR-Ab-positive gMG, the prespecified steroid-sparing outcome was achieved by 60% of rituximab-treated participants and 56% of those receiving placebo [53]. By contrast, in RINOMAX, which evaluated recent-onset gMG, 71% of rituximab-treated participants versus 29% receiving placebo achieved minimal disease manifestations at week 16 while receiving no more than 10 mg/day prednisolone and no rescue therapy [54]. Together with observational comparisons of new-onset and refractory disease, these contrasting results support the view that the clinical effect of B-cell depletion is context-dependent and may vary with disease stage, immune architecture, and the antibody-producing compartment [54,55].

Several mechanisms may therefore underlie incomplete RTX response in refractory AChR-Ab-positive gMG: persistence of mature plasma cells, pre-existing circulating IgG, re-emergent plasmablast or autoreactive B-cell populations, and less accessible tissue-resident immune compartments [49,50,56]. Broader B-lineage depletion refines, but does not abolish, this logic. Inebilizumab targets CD19-positive B-lineage cells, including a broader compartment than CD20-directed therapy, and the phase 3 MINT trial supports its clinical activity in antibody-positive gMG [15]. Even so, B-lineage targeting remains mechanistically distinct from FcRn inhibition, which reduces circulating IgG persistence, and from complement inhibition, which blocks downstream effector injury.

For sequencing, B-cell depletion is best understood as upstream control. It may reduce future autoantibody generation, but it does not neutralize antibodies already in circulation, block their binding at the NMJ, or prevent complement activation once pathogenic IgG is available. Persistent disease after RTX therefore raises a more precise question: is the active layer continued antibody production, residual circulating IgG, downstream complement injury, or a combination of these processes?

## 8. Complement Inhibition as Downstream Targeted Therapy in AChR-Ab-Positive Refractory Myasthenia Gravis

Complement activation is a major effector mechanism in AChR-Ab-positive gMG. Immunopathological and experimental studies demonstrate immune-complex deposition, complement components, and membrane attack complex at the motor endplate, supporting a causal role for complement-mediated postsynaptic injury [2,23]. This is one reason AChR-Ab-positive disease differs immunologically from MuSK-Ab-positive MG, which is often IgG4-dominant and less complement-driven [52].

Binding of AChR antibodies can activate the classical complement pathway, leading to C5 cleavage and C5b-9 membrane attack complex formation [2,23]. This biology provides the rationale for terminal complement inhibition. Randomized trials provide quantitative support for this strategy while also illustrating differences in trial design and statistical analysis. In REGAIN, the prespecified worst-rank ANCOVA analysis of the primary MG-ADL endpoint did not reach statistical significance (P = 0.0698); in a prespecified repeated-measures sensitivity analysis, the least-squares mean MG-ADL change at week 26 was −4.2 with eculizumab versus −2.3 with placebo [8]. In CHAMPION-MG, the least-squares mean change in MG-ADL at week 26 was −3.1 with ravulizumab versus −1.4 with placebo (between-group difference −1.6) [9]. In RAISE, the least-squares mean MG-ADL change at week 12 was −4.39 with zilucoplan versus −2.30 with placebo (difference −2.09, 95% CI −3.24 to −0.95) [10]. Extension studies further support durability of benefit in selected patients [57,58,59,60,61,62,63].

Evidence syntheses are useful but do not resolve sequencing. A 2025 Cochrane publication remains a protocol rather than a completed efficacy review, and a 2026 meta-analysis comparing complement inhibitors and FcRn blockers provides indirect comparative synthesis limited by differences in trial design, populations, background therapies, and endpoints [17,64]. The evidence therefore supports complement inhibition as an active mechanism, but not a fixed position in a post-failure treatment order.

The mechanistic boundary of C5 inhibition is central to the present review. C5 inhibitors act after autoantibody production and antibody binding: they block terminal complement activation but do not remove circulating pathogenic IgG, prevent AChR binding, suppress B-cell or plasma-cell compartments, or eliminate complement-independent antibody effects. Persistent symptoms during complement blockade may therefore reflect receptor blockade, receptor cross-linking, antigenic modulation, functional receptor loss, or continued IgG burden [2,23].

Safety also influences sequencing. Terminal complement blockade impairs defense against Neisseria meningitidis, and invasive meningococcal disease has been reported despite vaccination in eculizumab-treated patients [65]. Vaccination planning, prophylaxis considerations, counseling, infection risk, and urgency of treatment all become part of the decision, not secondary administrative details.

For mechanism-based sequencing, complement inhibition should be viewed as downstream effector control rather than global antibody-directed therapy. Partial benefit supports the clinical relevance of complement-mediated injury, but persistent activity after adequate C5 blockade should prompt reassessment of upstream IgG-mediated mechanisms. In selected patients with incomplete complement response and transient PLEX benefit, FcRn-mediated IgG reduction is a plausible mechanistic transition, although prospective switching data are still lacking [16,17,18].

## 9. FcRn Inhibition and Reduction in Circulating Pathogenic IgG Burden

FcRn is a central regulator of IgG homeostasis. By binding IgG within acidic endosomes and recycling it back to the circulation, FcRn protects IgG from intracellular degradation and prolongs serum IgG half-life. Experimental studies in beta2-microglobulin-deficient mice and early human pharmacology studies of FcRn blockade established that interfering with this pathway can produce rapid, reversible reductions in circulating IgG without directly suppressing IgG production [25,26].

This mechanism is directly relevant to AChR-Ab-positive gMG because pathogenic IgG antibodies contribute to weakness through receptor blockade, antigenic modulation with AChR loss, and complement activation at the NMJ [2,23]. FcRn inhibition lowers the circulating IgG pool available for these mechanisms. It does not eliminate autoreactive B cells, plasmablasts, or long-lived plasma cells; instead, it reduces the antibody burden generated by these compartments. This distinction matters when symptoms persist after B-cell-directed therapy, which may not rapidly clear pre-existing IgG or eliminate mature plasma cells [49,50].

In the therapeutic cascade, FcRn inhibition therefore occupies a different position from both upstream cellular therapy and downstream complement blockade. It can reduce the substrate for receptor binding, antigenic modulation, and complement activation while leaving the cellular sources of IgG production untouched. This is the reason FcRn blockade is mechanistically attractive in patients whose course suggests persistent circulating humoral pathogenicity.

Clinical evidence now supports FcRn inhibition as a therapeutic class in gMG, with quantitative efficacy demonstrated across several phase 3 programs. In ADAPT, 68% of AChR-Ab-positive participants receiving efgartigimod were MG-ADL responders during the first treatment cycle compared with 30% receiving placebo (odds ratio 4.95, 95% CI 2.21–11.53) [11]. In MycarinG, least-squares mean MG-ADL changes from baseline to day 43 were −3.37 and −3.40 with rozanolixizumab 7 mg/kg and 10 mg/kg, respectively, compared with −0.78 with placebo; the corresponding between-group differences were −2.59 and −2.62 [12]. In Vivacity-MG3, the least-squares mean change in MG-ADL from baseline across weeks 22–24 was −4.70 with nipocalimab versus −3.25 with placebo (difference −1.45, 95% CI −2.38 to −0.52) [13]. Long-term extension and repeated-exposure studies provide additional information regarding durability and safety [66,67,68,69]. Agent-specific differences in molecular format, dosing schedule, and exposure may be clinically relevant, but no head-to-head evidence establishes superiority of one FcRn inhibitor over another [11,12,13,14,70].

The class-level evidence is encouraging but should be read in context. A systematic review and meta-analysis found improvement across clinical and patient-reported outcomes compared with placebo without a clear increase in overall safety risk in pooled randomized trial data [71]. Network meta-analyses can place FcRn inhibitors within the broader landscape of innovative therapies, but they cannot define the optimal sequence after incomplete response to complement inhibition, B-lineage therapy, or conventional IS [16].

The sequencing value of FcRn inhibition follows from its target. Persistent disease after B-cell depletion may reflect pre-existing or plasma-cell-derived IgG; persistent disease after complement inhibition may reflect IgG-mediated mechanisms not fully neutralized by C5 blockade; and transient benefit from PLEX may keep circulating humoral factors clinically plausible. These observations are mechanistic inferences, not sequencing evidence, but they provide a coherent rationale for considering FcRn blockade when circulating IgG burden remains a credible driver.

Accordingly, FcRn inhibition should be framed in refractory AChR-Ab-positive gMG as an IgG-burden reduction strategy. It does not replace B-cell depletion, complement inhibition, IVIG, or PLEX; it addresses a different layer of the cascade. In selected patients, particularly those with transient benefit from antibody-removal therapy or incomplete control after cellular and downstream effector-directed treatments, FcRn blockade is a plausible next mechanism pending comparative and sequencing studies.

## 10. Mechanism-Based Sequencing in Refractory AChR-Ab-Positive Generalized Myasthenia Gravis

The expanded treatment landscape has shifted the question in refractory AChR-Ab-positive gMG from whether escalation is justified to how the next mechanism should be chosen. Guidelines support individualized treatment, but neither guidelines nor trials define a universal sequence among conventional IS, B-lineage therapy, complement inhibition, FcRn inhibition, IVIG, and PLEX after incomplete response to a previous mechanism [3,4,16,17,22]. A line-based model is therefore insufficient. Here, sequencing means using prior response, nonresponse, intolerance, CS dependence, rescue-therapy dependence, and respiratory vulnerability as clues to residual disease biology.

This approach is useful because the core processes in AChR-Ab-positive gMG—autoantibody generation, circulating IgG persistence, antibody binding at the NMJ, complement-mediated postsynaptic injury, and impaired neuromuscular transmission—are connected but therapeutically separable [1,2,23]. A partial or absent response rarely answers the entire pathophysiological question. More often, it poses a narrower one: which layer has not been adequately controlled? Figure 1 summarizes the cascade and therapeutic entry points; Table 1 translates these concepts into clinically reassessable response patterns.

At the bedside, the first distinction is between immediate instability and inadequate durable control. Bulbar weakness, dyspnea, impending crisis, or escalating ventilatory support require rapid stabilization, usually with IVIG or PLEX, while a longer-term disease-control strategy is developed [3,4,38,39,40,41,42,43]. By contrast, persistent CS dependence or moderate but functionally relevant weakness may allow time to optimize background steroid-sparing therapy before or alongside targeted escalation [3,4,24,27,28,29,30,31,32,33,34,35,36,37].

Before a response pattern is interpreted mechanistically, apparent treatment failure should first be confirmed as true and clinically meaningful. This includes reassessing the diagnosis and antibody subtype when appropriate, documenting objective MG activity, confirming agent-appropriate dose, exposure, duration, and adherence, and distinguishing persistent fatigable weakness from treatment toxicity, infection, comorbidity, fixed deficits, or deconditioning. Reasons for treatment interruption or intolerance should also be documented. Only after these competing explanations have been addressed should partial response or nonresponse be used as a clue to an unresolved pathogenic layer [3,4].

The prior response pattern then narrows the mechanistic hypothesis. Transient PLEX benefit supports continued humoral relevance; incomplete CD20-directed response may reflect residual plasma-cell-derived IgG; and partial C5-inhibitor benefit supports complement involvement while leaving complement-independent IgG effects possible [2,8,9,10,23,38,40,49,50,53,54,55,57,58,61,62,63]. These patterns do not identify a validated next step, but they justify reassessment of the unresolved pathogenic layer rather than simply labeling the patient globally refractory.

When circulating IgG remains plausible, FcRn inhibition is biologically coherent because it reduces IgG persistence pharmacologically rather than episodically removing plasma factors. Early pharmacodynamic work, pivotal trials, extension analyses, and meta-analytic data support this class as an IgG-lowering strategy in gMG [11,12,13,26,66,67,68,69,70,71]. These data support the mechanism; they do not establish a rule that FcRn inhibition must follow PLEX responsiveness or complement-inhibitor failure.

Safety, feasibility, and respiratory vulnerability may be as decisive as mechanism. Terminal complement inhibition requires meningococcal risk mitigation [65]. FcRn inhibition requires attention to total IgG reduction, infection risk, baseline immunoglobulins, repeated exposure, and concomitant IS [11,12,13,14,26,66,67,68,69,70,71]. B-cell and B-lineage therapies require attention to hypogammaglobulinemia, vaccine responses, infection risk, and immune reconstitution [15,49,50,53,54,55]. Route of administration, monitoring burden, cost, and access often determine which biologically plausible option is actually usable. In real-world practice, reimbursement or insurance authorization, local formulary restrictions, infusion-center capacity, travel requirements, and frequency of administration may delay or preclude a mechanistically preferred option; these financial and logistical constraints should therefore be treated as part of the sequencing decision rather than as separate administrative issues.

Validated biomarkers for choosing among C5 inhibition, FcRn inhibition, and B-lineage targeting are still lacking. AChR-Ab positivity supports the biological plausibility of complement inhibition and IgG-lowering therapy, but antibody status or titer alone does not identify the dominant mechanism in an individual refractory patient [1,2,23]. Until better biomarkers are available, clinical response patterns remain hypotheses supported by mechanism and trial data, not biomarker-defined rules.

Table 1 summarizes how common response patterns can be treated as mechanistic hypotheses and what should be reassessed before escalation or switching in refractory AChR-Ab-positive gMG.

In summary, mechanism-based sequencing is not an algorithm but a disciplined way to make escalation decisions explicit. Its value is to avoid two errors: treating every inadequate response as nonspecific severity, or treating a partial response or nonresponse as definitive proof for or against one mechanism. In refractory AChR-Ab-positive gMG, response patterns, intolerance, rescue dependence, and respiratory events should be interpreted alongside antibody subtype, disease stage, safety, feasibility, and patient-specific burden.

## 11. Illustrative Clinical Case

A 27-year-old woman developed intermittent binocular diplopia in 2017–2018, followed by bilateral ptosis, fluctuating limb weakness, and marked fatigability. Serum testing was positive for AChR-Ab, and chest computed tomography showed residual thymic tissue without thymoma. At early assessment, MG-ADL was approximately 5–6.

During the initial generalized phase, worsening symptoms required hospitalization and PLEX, which produced partial improvement. High-dose prednisone, up to 60 mg/day, and pyridostigmine were initiated. AZA was discontinued because of gastrointestinal intolerance and elevated liver enzymes. Over subsequent years, the disease remained active, with recurrent exacerbations, CS dependence, generalized weakness, ocular symptoms, fatigability, and intermittent dyspnea. A further exacerbation again improved partially after PLEX, with MG-ADL decreasing from approximately 9 to 4–5. RTX was administered at 800 mg every 3 months for three courses, but no sustained clinical benefit was observed. MTX was later added and titrated to 15 mg/week, while CS tapering remained limited by worsening.

The disease course was complicated by severe respiratory involvement. In 2022, severe coronavirus disease 2019 (COVID-19) was complicated by myasthenic crisis with respiratory failure, requiring approximately 2 months of mechanical ventilation, tracheostomy, and prolonged rehabilitation. During the crisis and recovery period, she received PLEX, IVIG, high-dose CS, and terminal complement inhibition with eculizumab. This was associated with partial improvement, but disease control remained incomplete, with continued dependence on IVIG and high-dose CS.

Eculizumab was subsequently switched to ravulizumab, administered every 8 weeks. This provided partial stabilization but did not eliminate disease activity or treatment dependence. Thymectomy was performed after clinical stabilization in 2023. A transient postoperative improvement was noted, but MG-ADL remained approximately 5 and the response was incomplete. Between 2023 and 2025, the patient continued ravulizumab, IVIG 40 g every 2 weeks, prednisone, and pyridostigmine. Attempts to extend IVIG intervals were followed by worsening. Fatigability, proximal weakness, intermittent dyspnea, and nocturnal bilevel positive airway pressure (BiPAP) requirement persisted, with MG-ADL remaining approximately 5–6.

By early 2026, the disease was considered severe refractory AChR-Ab-positive gMG, with persistent symptoms, CS dependence, IVIG dependence, respiratory vulnerability, and incomplete control despite PLEX, IVIG, high-dose CS, AZA intolerance, MTX, RTX, thymectomy, eculizumab, and ravulizumab. Before another PLEX course, MG-ADL was 12; after five PLEX procedures, MG-ADL improved to 6, but the effect was considered insufficient for durable control. This repeated but transient humoral rescue response contributed to the rationale for targeting persistence of circulating pathogenic IgG.

Nipocalimab was started in March 2026, with a loading dose of 3120 mg followed by 1560 mg every 2 weeks. MMF (CellCept) was recommended as planned background CS-sparing IS to reduce ongoing antibody production while nipocalimab reduced persistence of circulating IgG. This represented an individualized empirical strategy rather than a validated combination protocol. The patient did not initiate MMF for several months by her own decision, possibly because of concern about adverse effects.

For clarity, Table 2 summarizes the chronology of major treatments, clinical responses, and overlapping interventions relevant to interpretation of the case.

## 12. Mechanism-Based Discussion of the Clinical Case

This case illustrates severe refractory AChR-Ab-positive gMG with high disease burden: early CS dependence, recurrent exacerbations, IVIG dependence, respiratory involvement, crisis requiring prolonged ventilation and tracheostomy, and incomplete control despite interventions acting at several levels of the pathogenic cascade.

PLEX responsiveness was an important clue. Repeated partial responses, including MG-ADL improvement from 12 to 6 in early 2026, suggest that circulating humoral factors remained clinically relevant. Because the benefit was incomplete and transient, PLEX supported ongoing antibody-mediated disease activity rather than providing durable control.

The incomplete RTX response did not argue against antibody-mediated MG. CD20-directed therapy does not eliminate mature plasma cells or rapidly remove pre-existing circulating IgG [49,50]. BeatMG, RINOMAX, and observational data indicate that antibody subtype, timing, disease stage, and immune architecture all influence response to B-cell depletion [53,54,55]. In this patient, persistent symptoms after RTX were therefore compatible with continued plasma-cell-derived or circulating IgG-mediated activity.

The partial response to C5 inhibition was also informative. Randomized trials and extension studies validate terminal complement inhibition in AChR-Ab-positive gMG [8,9,10,57,58,61,62,63], and partial stabilization in this patient suggests that complement-mediated injury was clinically relevant. Persistent IVIG dependence, CS dependence, and respiratory vulnerability nevertheless indicated that C5 blockade alone did not control the full disease process.

Within this trajectory, FcRn inhibition was a coherent next mechanism to consider. FcRn blockade lowers circulating IgG through a pathway distinct from B-cell depletion and complement inhibition [25,26], and phase 3 evidence supports FcRn inhibition in gMG [11,12,13,69]. The rationale for nipocalimab came from the whole sequence: incomplete upstream control, incomplete downstream effector control, persistent rescue dependence, and recurrent humoral responsiveness.

The case should be interpreted cautiously and does not permit causal attribution to any single intervention. Early improvement followed both recent PLEX and nipocalimab initiation, while later improvement occurred during continued nipocalimab, MMF titration, temporary prednisone escalation, and supportive respiratory therapy. The natural fluctuation of MG further limits causal inference. Accordingly, this single-case trajectory cannot establish the superiority of FcRn inhibition over another therapeutic class, the safety or efficacy of a particular cross-class transition protocol, or a validated treatment sequence; it is presented to illustrate the reasoning process behind mechanism-based reassessment.

The proposed framework is intended to make reassessment before escalation explicit, not to rank drug classes. Because no prospective trial has established a universal sequence among C5 inhibition, FcRn inhibition, and B-lineage targeting, the practical task is to document the mechanistic hypothesis behind a switch and the evidence that would support or weaken it [3,4,16,18].

Reassessment begins by confirming that the apparent treatment failure is real and clinically relevant. Diagnosis and antibody subtype should be reconsidered when appropriate, and active fatigable weakness should be distinguished from fixed deficits, treatment toxicity, and comorbid conditions. Exposure, adherence, dose, duration, intolerance, and reason for discontinuation should be documented. The phenotype should then be described using symptom distribution, bulbar and respiratory involvement, MG-ADL and, where feasible, QMG or MGC, together with CS dose, rescue-therapy requirement, and functional burden [3,4,5,6,7,19,22].

Urgency should be considered separately from mechanism selection. Impending crisis, acute dyspnea, severe bulbar weakness, or rapidly worsening generalized weakness requires immediate stabilization, usually with IVIG or PLEX, while a durable strategy is planned [3,4,22,38,39,40,41,42,43]. In a clinically stable patient, there may be time to optimize background steroid-sparing therapy or to choose a targeted mechanism based on prior response and feasibility.

Prior responses should be interpreted as mechanistic clues rather than proof for or against a specific pathway; the general reassessment principles are summarized in Table 1.

The next mechanism should be both biologically coherent and feasible. Antibody subtype, disease stage, respiratory risk, comorbidities, infection history, immunoglobulin status, vaccination requirements, monitoring burden, access, and patient preference should be weighed alongside the mechanistic rationale. Pivotal targeted-therapy trials largely evaluated add-on treatment to standard care [8,9,10,11,12,13,14,15]; they do not define a universally optimal layered regimen or justify routine combination of targeted biologics.

Finally, success and failure should be defined before the change is made. Baseline MG-ADL, QMG or MGC where feasible, bulbar and respiratory status, CS dose, IVIG or PLEX frequency, hospitalizations, functional independence, and quality of life should be recorded. A short-term MG-ADL improvement is not durable control if respiratory instability, high CS exposure, or rescue-therapy dependence persists; conversely, a slower steroid-sparing agent should not be judged ineffective before adequate exposure [3,4,57,58,61,62,63,66,67,68,69,70].

At each reassessment, the working hypothesis, supporting evidence, safety constraints, and threshold for further change should be made explicit. This keeps sequencing revisable and testable while avoiding both automatic escalation and excessive confidence in any single response pattern.

## 13. Future Directions: From Mechanistic Inference to Testable Sequencing

### 13.1. Validating Treatment Sequencing and Predictive Biomarkers

Future progress in refractory AChR-Ab-positive gMG will depend not only on expanding the number of therapeutic options but on testing whether treatment-response patterns can be linked prospectively to patient-specific disease biology. Complement inhibition, FcRn-mediated IgG reduction, and B-lineage targeting have each demonstrated efficacy in randomized trials, yet current guidelines and comparative evidence do not provide a prospectively validated sequence after an incomplete response to another targeted mechanism [3,4,8,9,10,11,12,13,15,16,17,18]. Disease duration, thymic status, antibody subtype, prior immunotherapy, rescue-therapy dependence, respiratory vulnerability, and cumulative treatment burden may all modify the clinical value of a given mechanism. Emerging concepts such as MG age and disease stage should therefore be evaluated as potential modifiers of treatment response rather than used as established treatment-selection rules [72].

A major priority is the development of biomarkers capable of identifying the dominant pathogenic layer before a treatment change is made. Standard AChR-Ab and MuSK-Ab testing defines the best-established serological subgroups, but antibody status and titer alone do not reliably distinguish complement-mediated injury, circulating IgG burden, short-lived antibody-secreting cells, or long-lived plasma-cell activity in an individual patient. Longitudinal AChR-Ab dynamics, IgG kinetics during FcRn blockade, complement activity, B-cell and plasmablast signatures, and rescue-therapy dependence may be more informative when assessed together. Live or clustered cell-based assays can improve diagnostic classification in some seronegative patients, while LRP4 and other neuromuscular-junction-related antibodies may further refine biological subgroups; however, diagnostic enrichment should not be conflated with validated prediction of treatment response [72,73,74,75,76,77].

Future trials should therefore move beyond single-agent placebo-controlled efficacy and address predefined switching and sequencing questions. Relevant designs include FcRn inhibition after incomplete C5 inhibition, C5 inhibition after inadequate FcRn response, B-lineage targeting after persistent dependence on IgG-lowering therapy, and switching within a mechanistic class when route, dosing schedule, durability, or tolerability becomes limiting. Head-to-head randomized trials may not be feasible for every sequence, but prospective switching cohorts and harmonized registries could reduce the current dependence on indirect comparisons [16,17,18]. Outcomes should extend beyond short-term responder thresholds to sustained minimal symptom expression, freedom from rescue therapy, CS reduction, respiratory stability, cumulative treatment burden, and patient-reported stability [57,58,59,60,61,62,63,66,67,68,69,70,72].

### 13.2. Refining Established Mechanistic Classes

Development within established therapeutic classes is increasingly shifting from proof of efficacy toward convenience, durability, individualized exposure, and positioning within a broader treatment sequence. Complement inhibitors and FcRn inhibitors validate distinct components of the pathogenic cascade, but their within-class and cross-class positioning remains unsettled [8,9,10,11,12,13,14]. The phase 3 PREVAIL randomized trial has reported efficacy and safety data for once-weekly subcutaneous gefurulimab in AChR-Ab-positive gMG, supporting an additional complement-inhibition approach; however, as with other single-mechanism trials, these data do not define its position after incomplete response to another targeted mechanism [78]. For FcRn inhibitors, extension and sustained-control analyses support longer-term evaluation, but they do not establish interchangeability, optimal within-class switching, or which patients require continuous rather than cyclic IgG lowering [14,66,67,68,69,70,71].

Strategies targeting upstream B-lineage and plasma-cell biology may provide a different form of disease control by reducing cellular sources of pathogenic humoral immunity. The MINT trial supports CD19-positive B-lineage depletion with inebilizumab in gMG [15]. Dual BAFF/APRIL inhibition with povetacicept has reduced anti-AChR antibodies and disease activity in experimental autoimmune MG, providing a rationale for clinical development, although this evidence remains preclinical and cannot define a current sequencing role [79]. A central question for future studies is whether deeper upstream control can produce greater durability, reduce rescue-therapy dependence, and allow safer de-escalation of CS or conventional IS.

### 13.3. Emerging Mechanisms That May Extend the Sequencing Framework

These emerging approaches are relevant to the present framework not because they can yet be positioned within a clinical treatment sequence, but because they extend the same pathogenic map toward increasingly specific upstream, cellular, plasma-cell, and antigen-directed interventions. In this sense, future therapies may transform mechanism-based sequencing from inference based on treatment response into selection among progressively more precise levels of immune intervention.

Antigen-specific immune tolerance is attractive because it aims to modify autoimmune recognition while preserving broader protective immunity. CNP-106 is a biodegradable nanoparticle platform designed to deliver AChR-derived antigens in a tolerogenic context; the published first-in-human report describes the rationale and study design rather than established clinical efficacy [80]. Such approaches may ultimately help test whether disease stage, duration, and establishment of long-lived humoral memory determine the reversibility of pathogenic autoimmunity.

Cellular therapies have moved from conceptual interest to early clinical investigation. Descartes-08, an autologous BCMA-directed mRNA CAR-T therapy administered without lymphodepleting chemotherapy, and anti-BCMA/CD19 CAR-T therapy have both been evaluated in early MG studies [81,82]. These approaches raise a different sequencing question from currently available targeted biologics: whether a deeper intervention in antibody-producing cell compartments can provide durable disease control and reduce the need for repeated pharmacological suppression. Their clinical role will depend on durability, relapse biology, retreatment needs, immune reconstitution, infection risk, manufacturing access, and cost.

Antigen-specific cellular strategies seek still greater selectivity. MuSK-directed chimeric autoantibody receptor T cells and AChR-directed CAAR-T constructs have demonstrated antigen-specific activity in experimental systems [83,84]. These approaches aim to remove pathogenic autoreactive populations while limiting broader immune depletion, but clinical safety, durability, antigen selection, escape of autoreactive clones, and scalable manufacturing remain unresolved.

MGAC-007, described in a 2026 American Academy of Neurology abstract as an antigen-drug conjugate intended to sequester anti-AChR antibodies and eliminate AChR-reactive B-cell sources, provides another early translational example of antigen-directed immune editing [85]. BAFF/APRIL inhibition, antigen-specific tolerance, CAR-T, CAAR-T, and antigen-drug conjugates therefore represent different levels of intervention within the pathogenic cascade, but they remain at substantially different stages of evidentiary maturity and should be clearly distinguished from therapies supported by randomized clinical evidence [79,80,81,82,83,84,85].

### 13.4. Combination, De-Escalation, and Real-World Sequencing

Combination and de-escalation strategies represent another major evidence gap. Targeted therapies are commonly introduced on top of CS, conventional IS agents, IVIG, PLEX, or prior biologics, yet the optimal pace and sequence of background-therapy withdrawal remain uncertain. Prospective studies should distinguish additive efficacy from unnecessary cumulative immunosuppression and determine when CS, IVIG, PLEX, or conventional IS can be reduced safely [3,4,57,58,59,60,61,62,63,66,67,68,69,70]. Registries should capture antibody subtype, thymic status, disease duration, prior therapies, reasons for switching, rescue-therapy dependence, respiratory outcomes, CS exposure, biomarkers, patient-reported outcomes, adverse events, access constraints, and sustained response [18,72].

The next advance in refractory AChR-Ab-positive gMG is therefore unlikely to come from a longer linear treatment ladder alone. Progress will require prospective evidence for switching and de-escalation, validated biomarkers of pathogenic layers, durable patient-centered outcomes, and explicit separation of established clinical evidence from early translational promise. Current therapies provide distinct tools for IgG reduction, complement blockade, and B-lineage control; emerging tolerance, cellular, plasma-cell-directed, and antigen-specific approaches may eventually extend this framework toward more selective or durable modification of the pathogenic humoral compartment, but their place in mechanism-based sequencing remains to be established [72,79,80,81,82,83,84,85].

## 14. Limitations of Current Evidence and Mechanism-Based Inference

Several limitations should be considered when interpreting the proposed mechanism-based sequencing framework. Current evidence supports the efficacy of individual therapeutic mechanisms more strongly than it supports any specific sequence. Complement inhibitors, FcRn inhibitors, and CD19-directed B-lineage therapy were primarily tested against placebo or background standard care, not in trials designed to determine which mechanism should follow incomplete response to another targeted class [8,9,10,11,12,13,15]. Direct head-to-head comparisons remain scarce, and indirect or network comparisons are constrained by differences in antibody status, disease duration, background therapy, rescue-therapy rules, outcome definitions, and follow-up [16,17,18].

Refractory MG is defined inconsistently, with published criteria emphasizing different combinations of persistent symptoms, relapse during tapering, treatment intolerance, CS dependence, repeated IVIG or PLEX, crisis, or failure to achieve minimal manifestation status [5,6,7,19,20,21]. This heterogeneity is central to the present framework but limits generalizability. Clinical response patterns should therefore be interpreted as mechanistic hypotheses rather than biomarkers: improvement after PLEX supports continued relevance of circulating humoral factors but does not prove that pathogenic IgG is the only driver, and incomplete response to terminal complement inhibition does not demonstrate that complement is irrelevant [2,8,9,10,23,38,42,43,57,58,59,60,61,62,63].

The same caution applies to B-cell depletion and FcRn inhibition. Lack of sustained benefit from RTX does not exclude humoral autoimmunity because CD20-directed therapy does not directly remove pre-existing IgG or reliably eliminate mature plasma cells [49,50,53,54,55]. Improvement during FcRn inhibition supports the clinical relevance of IgG lowering, but does not establish superiority to complement inhibition, B-lineage targeting, or optimized conventional IS. Validated predictive biomarkers for selecting among targeted mechanisms are lacking; AChR-Ab status identifies a biologically coherent population for complement inhibition and IgG-lowering therapy, but antibody status or titer alone does not determine which pathogenic layer dominates in an individual patient [1,2,23,72,77].

The illustrative case is a single retrospective reconstruction intended to demonstrate a reasoning process rather than to establish efficacy or comparative superiority. MG-ADL was available, but QMG and MGC scores were not collected systematically. Attribution of improvement is limited by overlapping interventions, including recent PLEX, continued corticosteroids, delayed MMF initiation, respiratory support, and subsequent prednisone escalation. The chronology supports mechanistic plausibility, but it does not establish causality, a PLEX rebound, or nipocalimab as the preferred treatment after complement inhibition.

Safety, feasibility, access, and developmental maturity may constrain sequencing as strongly as pathophysiology. Terminal complement blockade requires meningococcal risk mitigation, FcRn inhibition requires attention to IgG reduction and infection during repeated exposure, and B-lineage therapy raises questions about vaccination, hypogammaglobulinemia, and immune reconstitution [15,65,66,67,68,69,70]. Future-directed strategies discussed in this review—including BAFF/APRIL inhibition, antigen-specific tolerance, CAR-T, CAAR-T, and antigen-drug conjugates—differ substantially in evidentiary maturity and should not be interpreted as equivalent to established randomized clinical evidence [79,80,81,82,83,84,85].

Despite these limitations, mechanism-based sequencing provides a transparent decision-support concept. Its value lies in integrating pathophysiology with treatment history, safety, feasibility, and patient-specific disease burden, while avoiding both nonspecific escalation and overinterpretation of any single response pattern. Its validity must ultimately be tested in prospective comparative, switching, biomarker, registry, and de-escalation studies [3,4,16,18].

## 15. Conclusions

Refractory AChR-Ab-positive generalized myasthenia gravis is more than failure of successive treatment lines. It is a high-burden clinical state shaped by persistent immune activity, CS dependence, treatment intolerance, recurrent exacerbations, respiratory vulnerability, rescue-therapy dependence, or incomplete control despite conventional and targeted immunotherapy. The key therapeutic question is therefore not only whether to escalate but what part of the pathogenic cascade remains insufficiently controlled.

Conventional IS, IVIG, PLEX, B-cell and B-lineage depletion, complement inhibition, and FcRn inhibition act at different levels of this cascade: broad immune suppression, short-term humoral stabilization, cellular sources of autoantibody generation, downstream complement-mediated injury, and persistence of circulating IgG. The contribution of the present approach is to read treatment history as a dynamic map of unresolved immunopathology rather than as a retrospective list of therapeutic failures.

The illustrative case shows how this reasoning can be applied. Incomplete control after conventional IS, rescue therapies, B-cell depletion, thymectomy, and terminal complement inhibition did not exclude ongoing antibody-mediated disease. Transient benefit from humoral rescue therapy, persistent CS and IVIG dependence, respiratory vulnerability, and partial response to complement blockade supported the hypothesis that circulating pathogenic IgG remained clinically relevant. In that context, FcRn inhibition was a coherent mechanism to consider.

This remains decision support, not a validated algorithm. The case does not prove the superiority of any therapy or sequence. Direct comparative trials, prospective switching studies, predictive biomarkers, and real-world registries are needed to determine how best to choose among FcRn inhibition, complement inhibition, B-lineage targeting, and future antigen-specific or cellular approaches. Until then, mechanism-based sequencing offers a structured way to integrate pathophysiology, prior response, safety, feasibility, and patient-specific disease burden.

## Figures and Tables

**Figure 1:**
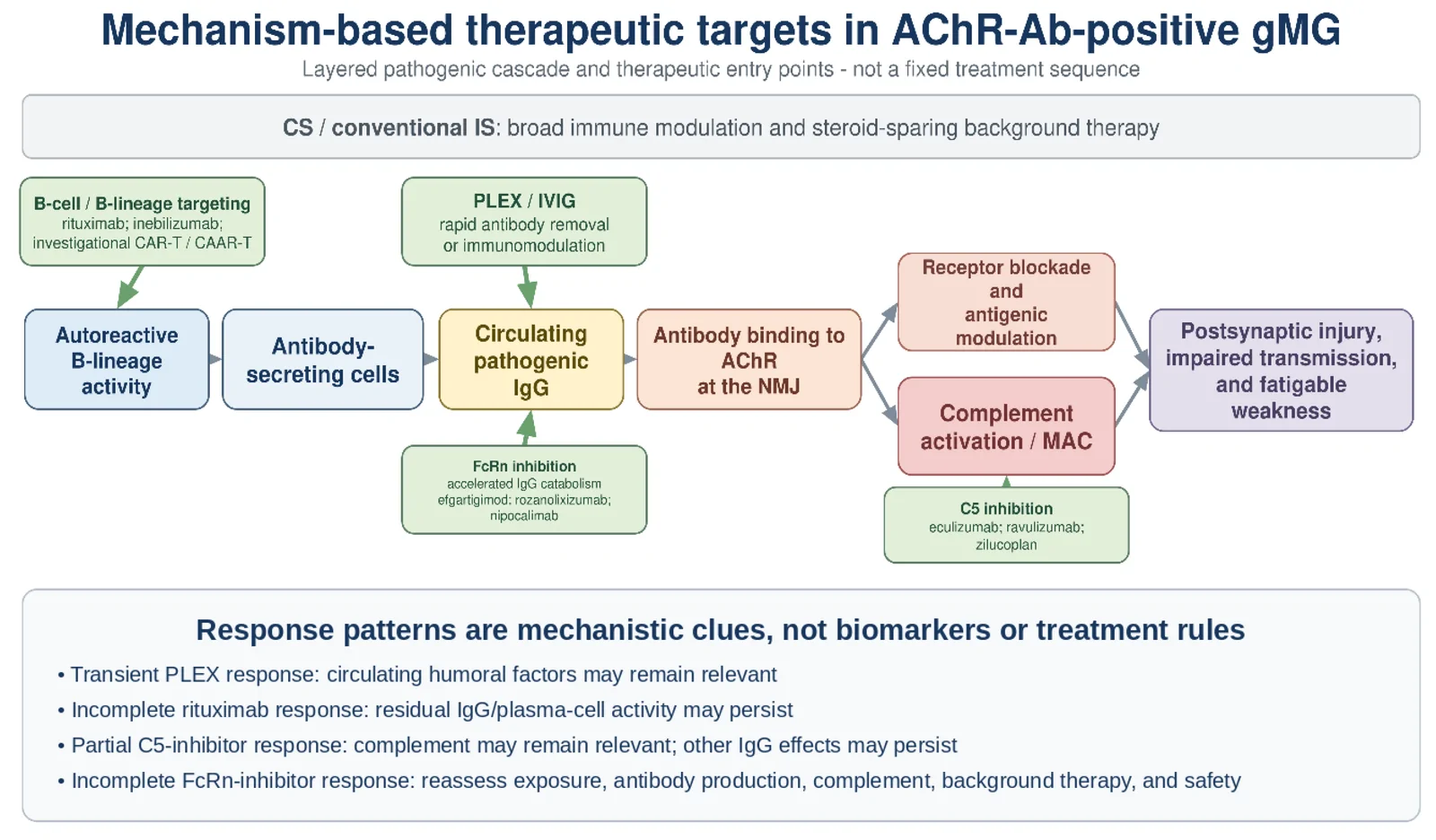
Mechanism-based therapeutic targets in acetylcholine receptor antibody-positive generalized myasthenia gravis. AChR-Ab-positive gMG can be conceptualized as a layered pathogenic cascade involving autoreactive B-lineage activity, differentiation into antibody-producing cells, persistence of circulating pathogenic IgG, antibody binding to AChR at the NMJ, receptor blockade and antigenic modulation, complement activation with MAC formation, and postsynaptic injury leading to fatigable weakness. Conventional CS and IS therapies broadly modulate immune activity; B-cell and B-lineage therapies target cellular sources or precursors of autoantibody generation; PLEX rapidly removes circulating plasma factors, whereas IVIG provides broader immunomodulation; FcRn inhibition accelerates IgG catabolism and lowers circulating IgG; and C5 inhibition blocks terminal complement activation and downstream postsynaptic injury. The central clinical message is that response patterns are mechanistic clues, not validated biomarkers or prescriptive treatment rules. Abbreviations: AChR, acetylcholine receptor; AChR-Ab, acetylcholine receptor antibody; C5, complement component 5; CAAR-T, chimeric autoantibody receptor T-cell; CAR-T, chimeric antigen receptor T-cell; CD20, cluster of differentiation 20; CS, corticosteroids; FcRn, neonatal Fc receptor; gMG, generalized myasthenia gravis; IgG, immunoglobulin G; IS, immunosuppressive; IVIG, intravenous immunoglobulin; MAC, membrane attack complex; NMJ, neuromuscular junction; PLEX, plasma exchange; RTX, rituximab.

**Table 1:** Clinical response patterns: mechanistic hypotheses and reassessment before treatment changes.

Clinical Pattern	Mechanistic Hypothesis	Reassess Before Treatment Change
Persistent symptoms or corticosteroid dependence despite conventional immunosuppressive (IS) therapy	Upstream immune activity remains inadequately controlled; conventional IS agents may have delayed onset, incomplete efficacy, or poor tolerability.	Confirm dose, exposure, and duration. Optimize steroid-sparing therapy when feasible; consider targeted escalation if clinically significant weakness, corticosteroid or rescue-therapy dependence, or instability persists.
Transient response to PLEX and/or IVIG	PLEX response supports continued relevance of circulating humoral factors; IVIG response supports reversible immune-mediated dysfunction but is less mechanism-specific. Durable control remains incomplete.	Confirm response duration, rescue frequency, respiratory status, and background therapy. Neither pattern predicts response to a specific targeted class.
Incomplete response to CD20-directed therapy (e.g., rituximab)	Pre-existing pathogenic IgG or active antibody production by long-lived plasma cells or plasmablasts persists despite peripheral CD20+ B-cell depletion.	Review the timing of administration, the achieved degree of B-cell depletion, baseline immunoglobulins, disease stage, and antibody subtype (AChR vs. MuSK). Nonresponse does not exclude an antibody-mediated disease model.
Partial response to terminal complement (C5) inhibition	Complement-mediated injury remains relevant, but complement-independent IgG effects (receptor blockade and antigenic modulation) may persist.	Verify exposure, adherence, clinical phenotype, and objective response metrics. Partial benefit does not automatically define FcRn inhibition as the next step.
Initial response followed by worsening during FcRn inhibition	IgG reduction may be insufficient; consider ongoing antibody production, exposure patterns, background therapy, infection, or comorbidities.	Reassess MG-ADL (and QMG/MGC where feasible), dosing adherence, total IgG, concomitant therapy, infection status, and serum albumin when clinically indicated or recommended for the specific agent. Worsening warrants reassessment of the overall disease-control strategy, including background therapy, rather than automatic class switching.
Persistent dependency on corticosteroids, IVIG, PLEX, or respiratory support	Durable disease control remains inadequate despite short-term or episodic improvement.	Quantify treatment and functional burden. Select the next long-term mechanism using antibody subtype, prior response, safety, feasibility, and patient preference.
Acute instability (impending or manifest crisis, acute dyspnea, severe bulbar weakness, or escalating ventilatory support)	High-risk disease requiring urgent stabilization before longer-term sequencing.	Prioritize PLEX or IVIG with respiratory/bulbar monitoring; select durable maintenance after acute risk is controlled.
Major safety or physiological constraints (infection risk, hypogammaglobulinemia, pregnancy, renal disease, or frailty)	Safety, tolerability, and physiological constraints may override the theoretically preferred mechanism.	Screen baseline immunoglobulins, vaccination status, and organ function. Prioritize safety and feasibility over mechanistic fit when necessary.

Abbreviations: AChR-Ab, acetylcholine receptor antibody; C5, complement component 5; CD20, cluster of differentiation 20; FcRn, neonatal Fc receptor; IgG, immunoglobulin G; IS, immunosuppressive; IVIG, intravenous immunoglobulin; MG, myasthenia gravis; MG-ADL, Myasthenia Gravis Activities of Daily Living; MGC, Myasthenia Gravis Composite; MuSK, muscle-specific kinase; PLEX, plasma exchange; QMG, Quantitative Myasthenia Gravis.

**Table 2:** Chronology of major treatments and clinical responses in the illustrative case.

Period	Treatment and Clinical Context	Response/Interpretive Relevance
2017–2018	Onset with diplopia, ptosis, generalized fatigable weakness; AChR-Ab-positive. Initial hospitalization with PLEX; prednisone up to 60 mg/day and pyridostigmine. AZA discontinued for gastrointestinal and hepatic intolerance.	MG-ADL approximately 5–6 at early assessment; partial improvement after PLEX.
Subsequent years before 2022	Recurrent exacerbations and CS dependence. Repeated PLEX; RTX 800 mg every 3 months for three courses; MTX titrated to 15 mg/week.	PLEX again produced partial benefit (MG-ADL approximately 9 to 4–5); RTX produced no sustained clinical benefit; CS taper remained limited by worsening.
2022	Severe COVID-19 complicated by myasthenic crisis with respiratory failure, prolonged mechanical ventilation and tracheostomy. PLEX, IVIG, high-dose CS, and eculizumab were used during crisis/recovery.	Partial improvement, but durable control remained incomplete with continued IVIG and high-dose CS dependence.
2022–2023	Eculizumab was switched to ravulizumab every 8 weeks; thymectomy was performed after stabilization in 2023.	Partial stabilization on complement inhibition; transient postoperative improvement, with MG-ADL remaining approximately 5.
2023–2025	Ravulizumab, IVIG 40 g every 2 weeks, prednisone, pyridostigmine, and nocturnal BiPAP as needed.	MG-ADL approximately 5–6; attempts to extend IVIG intervals were followed by worsening, indicating persistent treatment dependence.
Early 2026	Five-procedure PLEX course before transition to FcRn-directed therapy.	MG-ADL improved from 12 to 6, but the benefit was incomplete and transient.
March 2026	Nipocalimab started (3120 mg loading dose, then 1560 mg every 2 weeks). MMF was recommended as background CS-sparing therapy but was not initiated for several months.	The therapeutic change was individualized and empirical rather than a validated combination or switching protocol.
2026 follow-up	Continued nipocalimab; MMF was later initiated/titrated and prednisone was temporarily escalated; respiratory support remained part of the clinical context.	Clinical improvement occurred during overlapping interventions. The temporal sequence does not permit causal attribution to nipocalimab or establish superiority or safety of a specific cross-class transition.

## Data Availability

No new datasets were generated or analyzed in this study. Data sharing is not applicable to this narrative review. Additional case details are not publicly available in order to protect patient privacy.

## Abbreviations

AChR, acetylcholine receptor; AChR-Ab, acetylcholine receptor antibody; ANCOVA, analysis of covariance; APRIL, a proliferation-inducing ligand; AZA, azathioprine; BAFF, B-cell activating factor; BCMA, B-cell maturation antigen; BiPAP, bilevel positive airway pressure; C5, complement component 5; CAAR-T, chimeric autoantibody receptor T-cell; CAR-T, chimeric antigen receptor T-cell; CD19, cluster of differentiation 19; CD20, cluster of differentiation 20; CI, confidence interval; COVID-19, coronavirus disease 2019; CS, corticosteroids; FcRn, neonatal Fc receptor; gMG, generalized myasthenia gravis; IgG, immunoglobulin G; IS, immunosuppressive therapy; IVIG, intravenous immunoglobulin; LRP4, low-density lipoprotein receptor-related protein 4; MAC, membrane attack complex; MG, myasthenia gravis; MG-ADL, Myasthenia Gravis Activities of Daily Living; MGC, Myasthenia Gravis Composite; MMF, mycophenolate mofetil; MTX, methotrexate; MuSK, muscle-specific kinase; NMJ, neuromuscular junction; PLEX, plasma exchange; QMG, Quantitative Myasthenia Gravis; RTX, rituximab.

## References

[1] Gilhus N.E., Tzartos S., Evoli A., Palace J., Burns T.M., Verschuuren J.J.G.M. (2019). Myasthenia gravis. Nat. Rev. Dis. Primers.

[2] Gilhus N.E., Skeie G.O., Romi F., Lazaridis K., Zisimopoulou P., Tzartos S. (2016). Myasthenia gravis-autoantibody characteristics and their implications for therapy. Nat. Rev. Neurol..

[3] Narayanaswami P., Sanders D.B., Wolfe G., Benatar M., Cea G., Evoli A., Gilhus N.E., Illa I., Kuntz N.L., Massey J.M. (2021). International Consensus Guidance for Management of Myasthenia Gravis: 2020 Update. Neurology.

[4] Shelly S., Gotkine M., Wilf Yarkoni A., Lotan I., Abraham A., Dori A., Wolfe G.I., Regev K., Vaknin A., Ben-Hur T. (2025). National guidelines for diagnosis, treatment, and management of myasthenia gravis in Israel. Ther. Adv. Neurol. Disord..

[5] Suh J., Goldstein J.M., Nowak R.J. (2013). Clinical characteristics of refractory myasthenia gravis patients. Yale J. Biol. Med..

[6] Mantegazza R., Antozzi C. (2018). When myasthenia gravis is deemed refractory: Clinical signposts and treatment strategies. Ther. Adv. Neurol. Disord..

[7] Tran C., Biswas A., Mendoza M., Katzberg H., Bril V., Barnett C. (2021). Performance of different criteria for refractory myasthenia gravis. Eur. J. Neurol..

[8] Howard J.F., Utsugisawa K., Benatar M., Murai H., Barohn R.J., Illa I., Jacob S., Vissing J., Burns T.M., Kissel J.T. (2017). Safety and efficacy of eculizumab in anti-acetylcholine receptor antibody-positive refractory generalized myasthenia gravis (REGAIN): A phase 3, randomized, double-blind, placebo-controlled, multicentre study. Lancet Neurol..

[9] Vu T., Meisel A., Mantegazza R., Annane D., Katsuno M., Aguzzi R., Enayetallah A., Beasley K.N., Rampal N., Howard J.F. (2022). Terminal complement inhibitor ravulizumab in generalized myasthenia gravis. NEJM Evid..

[10] Howard J.F., Bresch S., Genge A., Hewamadduma C., Hinton J., Hussain Y., Kaminski H.J., Mantegazza R., Niks E.H., Raison J. (2023). Safety and efficacy of zilucoplan in patients with generalized myasthenia gravis (RAISE): A randomized, double-blind, placebo-controlled, phase 3 study. Lancet Neurol..

[11] Howard J.F., Bril V., Vu T., Karam C., Peric S., Margania T., Murai H., Bilinska M., Shakarishvili R., Smilowski M. (2021). Safety, efficacy, and tolerability of efgartigimod in patients with generalized myasthenia gravis (ADAPT): A multicentre, randomized, placebo-controlled, phase 3 trial. Lancet Neurol..

[12] Bril V., Druzdz A., Grosskreutz J., Habib A.A., Mantegazza R., Sacconi S., Utsugisawa K., Vissing J., Vu T., Boehnlein M. (2023). Safety and efficacy of rozanolixizumab in patients with generalized myasthenia gravis (MycarinG): A randomized, double-blind, placebo-controlled, adaptive phase 3 study. Lancet Neurol..

[13] Antozzi C., Beydoun S.R., Nowak R.J., Katzberg H., Ait-Tihyaty M., Gandhi K., Sheehan J.J., Pease S., Turkoz I., Sattin B. (2025). Safety and efficacy of nipocalimab in adults with generalized myasthenia gravis (Vivacity-MG3): A phase 3, randomized, double-blind, placebo-controlled study. Lancet Neurol..

[14] Howard J.F., Vu T., Li G., Korobko D., Smilowski M., Liu L., Gistelinck F., Steeland S., Noukens J., Van Hoorick B. (2024). Subcutaneous efgartigimod PH20 in generalized myasthenia gravis: A phase 3 randomized noninferiority study (ADAPT-SC) and interim analyses of a long-term open-label extension study (ADAPT-SC+). Neurotherapeutics.

[15] Nowak R.J., Benatar M., Ciafaloni E., Howard J.F., Leite M.I., Utsugisawa K., Vissing J., Rojavin M., Li Q., Tang F. (2025). A phase 3 trial of inebilizumab in generalized myasthenia gravis. N. Engl. J. Med..

[16] Sacca F., Pane C., Espinosa P.E., Sormani M.P., Signori A. (2023). Efficacy of innovative therapies in myasthenia gravis: A systematic review, meta-analysis and network meta-analysis. Eur. J. Neurol..

[17] Filippakopoulou E., Gavriilaki M., Arnaoutoglou M., Kimiskidis V., Giovanni S.D. (2026). Efficacy and safety of complement inhibitors and FcRn blockers in generalized AChR antibody-positive myasthenia gravis: A meta-analysis. J. Neurol..

[18] Huntemann N., Gerischer L.M., Herdick M., Nelke C., Stascheit F., Hoffmann S., Öztürk M., Schroeter C.B., Lehnerer S., Stein M. (2025). C5 complement inhibition versus FcRn modulation in generalised myasthenia gravis. J. Neurol. Neurosurg. Psychiatry.

[19] Cortes-Vicente E., Alvarez-Velasco R., Pla-Junca F., Rojas-Garcia R., Paradas C., Sevilla T., Casasnovas C., Gómez-Caravaca M.T., Pardo J., Ramos-Fransi A. (2022). Drug-refractory myasthenia gravis: Clinical characteristics, treatments, and outcome. Ann. Clin. Transl. Neurol..

[20] Rath J., Brunner I., Tomschik M., Zulehner G., Hilger E., Krenn M., Paul A., Cetin H., Zimprich F. (2020). Frequency and clinical features of treatment-refractory myasthenia gravis. J. Neurol..

[21] Jeong S., Noh Y., Oh I.S., Hong Y.H., Shin J.Y. (2021). Survival, prognosis, and clinical feature of refractory myasthenia gravis: A 15-year nationwide cohort study. J. Korean Med. Sci..

[22] Deutsche Gesellschaft für Neurologie (DGN) Diagnostik und Therapie Myasthener Syndrome: S2k-Leitlinie; AWMF-Registernummer 030-087; Version January 2025. https://register.awmf.org/assets/guidelines/030-087l_S2k_Diagnostik-Therapie-myasthener-Syndrome_2025-01.pdf.

[23] Phillips W.D., Vincent A. (2016). Pathogenesis of myasthenia gravis: Update on disease types, models, and mechanisms. F1000Research.

[24] Palace J., Newsom-Davis J., Lecky B. (1998). A randomized double-blind trial of prednisolone alone or with azathioprine in myasthenia gravis. Neurology.

[25] Israel E.J., Wilsker D.F., Hayes K.C., Schoenfeld D., Simister N.E. (1996). Increased clearance of IgG in mice that lack beta 2-microglobulin: Possible protective role of FcRn. Immunology.

[26] Ling L.E., Hillson J.L., Tiessen R.G., Bosje T., van Iersel M.P., Nix D.J., Markowitz L., Cilfone N.A., Duffner J., Streisand J.B. (2019). M281, an anti-FcRn antibody: Pharmacodynamics, pharmacokinetics, and safety across the full range of IgG reduction in a first-in-human study. Clin. Pharmacol. Ther..

[27] Pasnoor M., He J., Herbelin L., Burns T.M., Nations S.P., Bril V., Kissel J.T., Wolfe G.I., McVey A.L., Saperstein D.S. (2016). A randomized controlled trial of methotrexate for patients with generalized myasthenia gravis. Neurology.

[28] Di L., Shen F., Wen X., Lu Y., Zhu W., Wang M., Da Y. (2022). A randomized open-labeled trial of methotrexate as a steroid-sparing agent in generalized myasthenia gravis. Front. Immunol..

[29] Tindall R.S., Rollins J.A., Phillips J.T., Greenlee R.G., Wells L., Belendiuk G. (1987). Preliminary results of a double-blind, randomized, placebo-controlled trial of cyclosporine in myasthenia gravis. N. Engl. J. Med..

[30] Tindall R.S., Phillips J.T., Rollins J.A., Wells L., Hall K. (1993). A clinical therapeutic trial of cyclosporine in myasthenia gravis. Ann. N. Y. Acad. Sci..

[31] Yoshikawa H., Kiuchi T., Saida T., Takamori M. (2011). Randomized, double-blind, placebo-controlled study of tacrolimus in myasthenia gravis. J. Neurol. Neurosurg. Psychiatry.

[32] Sanders D.B., Hart I.K., Mantegazza R., Shukla S.S., Siddiqi Z.A., De Baets M.H., Melms A., Nicolle M.W., Solomons N., Richman D.P. (2008). An international, phase III, randomized trial of mycophenolate mofetil in myasthenia gravis. Neurology.

[33] Muscle Study Group (2008). A trial of mycophenolate mofetil with prednisone as initial immunotherapy in myasthenia gravis. Neurology.

[34] Narayanaswami P., Sanders D.B., Thomas L., Thibault D., Blevins J., Desai R., Krueger A., Bibeau K., Liu B., Guptill J.T. (2024). Comparative effectiveness of azathioprine and mycophenolate mofetil for myasthenia gravis (PROMISE-MG): A prospective cohort study. Lancet Neurol..

[35] Sharshar T., Porcher R., Demeret S., Tranchant C., Gueguen A., Eymard B., Nadaj-Pakleza A., Spinazzi M., Grimaldi L., Birnbaum S. (2021). Comparison of corticosteroid tapering regimens in myasthenia gravis: A randomized clinical trial. JAMA Neurol..

[36] Schneider-Gold C., Gajdos P., Toyka K.V., Hohlfeld R.R. (2005). Corticosteroids for myasthenia gravis. Cochrane Database Syst. Rev..

[37] Wiendl H., Murai H., Narayanaswami P., Sacca F., Silvestri N.J., Howard J.F. (2025). Appropriate use of steroids for patients with generalized myasthenia gravis: An international Delphi study. Ther. Adv. Neurol. Disord..

[38] Pinching A.J., Peters D.K. (1976). Remission of myasthenia gravis following plasma-exchange. Lancet.

[39] Gajdos P., Chevret S., Clair B., Tranchant C., Chastang C. (1997). Clinical trial of plasma exchange and high-dose intravenous immunoglobulin in myasthenia gravis. Ann. Neurol..

[40] Dalakas M.C., Meisel A. (2022). Immunomodulatory effects and clinical benefits of intravenous immunoglobulin in myasthenia gravis. Expert Rev. Neurother..

[41] Barth D., Nabavi Nouri M., Ng E., Nwe P., Bril V. (2011). Comparison of IVIg and PLEX in patients with myasthenia gravis. Neurology.

[42] Ipe T.S., Davis A.R., Raval J.S. (2021). Therapeutic plasma exchange in myasthenia gravis: A systematic literature review and meta-analysis of comparative evidence. Front. Neurol..

[43] Pavlekovics M., Engh M.A., Lugosi K., Szabo L., Hegyi P., Terebessy T., Csukly G., Molnar Z., Illes Z., Lovas G. (2023). Plasma exchange versus intravenous immunoglobulin in worsening myasthenia gravis: A systematic review and meta-analysis with special attention to faster relapse control. Biomedicines.

[44] Bril V., Szczudlik A., Vaitkus A., Rozsa C., Kostera-Pruszczyk A., Hon P., Bednarik J., Tyblova M., Köhler W., Toomsoo T. (2023). A randomized, double-blind, placebo-controlled trial of the corticosteroid-sparing effects of immunoglobulin in myasthenia gravis. Neurology.

[45] Bril V., Berkowicz T., Szczudlik A., Nicolle M.W., Bednarik J., Hon P., Vaitkus A., Vu T., Rozsa C., Magnus T. (2025). Efficacy and safety of maintenance intravenous immunoglobulin in generalized myasthenia gravis patients with acetylcholine receptor antibodies: A multicenter, double-blind, placebo-controlled trial. Muscle Nerve.

[46] Manolopoulos A., Alzuabi M., Elmashala A., Cashwell J., Murad M.H., Naddaf E. (2025). Immunoglobulin for myasthenia gravis. Cochrane Database Syst. Rev..

[47] Yi J.S., Guptill J.T., Stathopoulos P., Nowak R.J., O’Connor K.C. (2018). B cells in the pathophysiology of myasthenia gravis. Muscle Nerve.

[48] Huda R. (2020). New approaches to targeting B cells for myasthenia gravis therapy. Front. Immunol..

[49] Hofmann K., Clauder A.K., Manz R.A. (2018). Targeting B cells and plasma cells in autoimmune diseases. Front. Immunol..

[50] Hiepe F., Dorner T., Hauser A.E., Hoyer B.F., Mei H., Radbruch A. (2011). Long-lived autoreactive plasma cells drive persistent autoimmune inflammation. Nat. Rev. Rheumatol..

[51] Hehir M.K., Hobson-Webb L.D., Benatar M., Barnett C., Silvestri N.J., Howard J.F., Howard D., Visser A., Crum B.A., Nowak R. (2017). Rituximab as treatment for anti-MuSK myasthenia gravis: Multicenter blinded prospective review. Neurology.

[52] Huijbers M.G., Zhang W., Klooster R., Niks E.H., Friese M.B., Straasheijm K.R., Thijssen P.E., Vrolijk H., Plomp J.J., Vogels P. (2013). MuSK IgG4 autoantibodies cause myasthenia gravis by inhibiting binding between MuSK and Lrp4. Proc. Natl. Acad. Sci. USA.

[53] Nowak R.J., Coffey C.S., Goldstein J.M., Dimachkie M.M., Benatar M., Kissel J.T., Wolfe G.I., Burns T.M., Freimer M.L., Nations S. (2022). Phase 2 trial of rituximab in acetylcholine receptor antibody-positive generalized myasthenia gravis: The BeatMG study. Neurology.

[54] Piehl F., Eriksson-Dufva A., Budzianowska A., Feresiadou A., Hansson W., Hietala M.A., Håkansson I., Johansson R., Jons D., Kmezic I. (2022). Efficacy and safety of rituximab for new-onset generalized myasthenia gravis: The RINOMAX randomized clinical trial. JAMA Neurol..

[55] Brauner S., Eriksson-Dufva A., Hietala M.A., Frisell T., Press R., Piehl F. (2020). Comparison between rituximab treatment for new-onset generalized myasthenia gravis and refractory generalized myasthenia gravis. JAMA Neurol..

[56] Stathopoulos P., Kumar A., Nowak R.J., O’Connor K.C. (2017). Autoantibody-producing plasmablasts after B cell depletion identified in muscle-specific kinase myasthenia gravis. JCI Insight.

[57] Muppidi S., Utsugisawa K., Benatar M., Murai H., Barohn R.J., Illa I., Jacob S., Vissing J., Burns T.M., Kissel J.T. (2019). Long-term safety and efficacy of eculizumab in generalized myasthenia gravis. Muscle Nerve.

[58] Mantegazza R., Wolfe G.I., Muppidi S., Wiendl H., Fujita K.P., O’Brien F.L., Booth H.D., Howard J.F. (2021). Post-intervention status in patients with refractory myasthenia gravis treated with eculizumab during REGAIN and its open-label extension. Neurology.

[59] Siddiqi Z.A., Nowak R.J., Mozaffar T., O’BRien F., Yountz M., Patti F., the REGAIN Study Group (2021). Eculizumab in refractory generalized myasthenia gravis previously treated with rituximab: Subgroup analysis of REGAIN and its extension study. Muscle Nerve.

[60] Jacob S., Murai H., Utsugisawa K., Nowak R.J., Wiendl H., Fujita K.P., O’brien F., Howard J.J.F. (2020). Response to eculizumab in patients with myasthenia gravis recently treated with chronic IVIg: A subgroup analysis of REGAIN and its open-label extension study. Ther. Adv. Neurol. Disord..

[61] Meisel A., Annane D., Vu T., Mantegazza R., Katsuno M., Aguzzi R., Frick G., Gault L., Howard J.F., the CHAMPION MG Study Group (2023). Long-term efficacy and safety of ravulizumab in adults with anti-acetylcholine receptor antibody-positive generalized myasthenia gravis: CHAMPION MG open-label extension. J. Neurol..

[62] Vu T.H., Mantegazza R., Annane D., Katsuno M., Meisel A., Nicolle M.W., Bril V., Aguzzi R., Frick G., Howard J.F. (2025). Long-term efficacy and safety of ravulizumab in adults with anti-acetylcholine receptor antibody-positive generalized myasthenia gravis: Final CHAMPION MG open-label extension results. Eur. J. Neurol..

[63] Howard J.F., Bresch S., Farmakidis C., Freimer M., Genge A., Hewamadduma C., Hinton J., Hussain Y., Juntas-Morales R., Kaminski H.J. (2024). Long-term safety and efficacy of zilucoplan in patients with generalized myasthenia gravis: Interim analysis of the RAISE-XT open-label extension study. Ther. Adv. Neurol. Disord..

[64] White L.M., Clay F.J., Forbes A.-M., Keh R.Y.S., Lilleker J.B., Spillane J., Storms K., Sussman J., Dodd K.C. (2025). Complement inhibitors for myasthenia gravis in adults (Protocol). Cochrane Database Syst. Rev..

[65] McNamara L.A., Topaz N., Wang X., Hariri S., Fox L., MacNeil J.R. (2017). High risk for invasive meningococcal disease among patients receiving eculizumab (Soliris) despite receipt of meningococcal vaccine. MMWR Morb. Mortal. Wkly. Rep..

[66] Howard J.F., Bril V., Vu T., Karam C., Peric S., De Bleecker J.L., Murai H., Meisel A., Beydoun S.R., Pasnoor M. (2024). Long-term safety, tolerability, and efficacy of efgartigimod (ADAPT+): Interim results from a phase 3 open-label extension study in participants with generalized myasthenia gravis. Front. Neurol..

[67] Habib A.A., Drużdż A., Grosskreutz J., Mantegazza R., Sacconi S., Utsugisawa K., Vu T., Vissing J., Gayfieva M., Pulido-Valdeolivas I. (2025). Long-term safety of cyclical rozanolixizumab in patients with generalized myasthenia gravis. J. Neuromuscul. Dis..

[68] Bril V., Druzdz A., Grosskreutz J., Habib A.A., Mantegazza R., Sacconi S., Utsugisawa K., Vu T., Boehnlein M., Greve B. (2025). Rozanolixizumab in generalized myasthenia gravis: Pooled analysis of the phase 3 MycarinG study and two open-label extensions. J. Neuromuscul. Dis..

[69] Vu T., Beydoun S.R., Nowak R.J., Katzberg H., Ait-Tihyaty M., Gandhi K., Sheehan J.J., Pease S., Turkoz I., Sattin B. (2026). Evaluation of sustained disease control with nipocalimab versus placebo in the phase 3 Vivacity-MG3 study. Eur. J. Neurol..

[70] Seth N.P., Xu R., DuPrie M., Choudhury A., Sihapong S., Tyler S., Meador J., Avery W., Cochran E., Daly T. (2025). Nipocalimab, an immunoselective FcRn blocker that lowers IgG and has unique molecular properties. mAbs.

[71] Li J., Wu X., Chu T., Tan X., Wang S., Qu R., Chen Z., Wang Z. (2024). The efficacy and safety of FcRn inhibitors in patients with myasthenia gravis: A systematic review and meta-analysis. J. Neurol..

[72] Binks S.N.M., Morse I.M., Ashraghi M., Vincent A., Waters P., Leite M.I. (2025). Myasthenia gravis in 2025: Five new things and four hopes for the future. J. Neurol..

[73] Damato V., Spagni G., Monte G., Woodhall M., Jacobson L., Falso S., Smith T., Iorio R., Waters P., Irani S.R. (2022). Clinical value of cell-based assays in the characterisation of seronegative myasthenia gravis. J. Neurol. Neurosurg. Psychiatry.

[74] Pevzner A., Schoser B., Peters K., Cosma N.C., Karakatsani A., Schalke B., Melms A., Kroger S. (2012). Anti-LRP4 autoantibodies in AChR- and MuSK-antibody-negative myasthenia gravis. J. Neurol..

[75] Rivner M.H., Quarles B.M., Pan J.X., Yu Z., Howard J.F., Corse A., Dimachkie M.M., Jackson C., Vu T., Small G. (2020). Clinical features of LRP4/agrin-antibody-positive myasthenia gravis: A multicenter study. Muscle Nerve.

[76] Gallardo E., Martinez-Hernandez E., Titulaer M.J., Huijbers M.G., Martínez M.A., Ramos A., Querol L., Díaz-Manera J., Rojas-García R., Hayworth C.R. (2014). Cortactin autoantibodies in myasthenia gravis. Autoimmun. Rev..

[77] Fichtner M.L., Horstkorte L., Sanchez Navarro B.G., Schmidt H., Cabraal I., Waters P.J., Leite M.I. (2025). Autoantibodies in myasthenia gravis. Int. Rev. Neurobiol..

[78] Gwathmey K.G., Sacca F., Howard J.F., Vu T., Xi J., Maurer M., Ruck T., Shin H.Y., Takahashi M.P., Casasnovas C. (2026). Efficacy and safety of gefurulimab in generalized myasthenia gravis: The PREVAIL phase 3 randomized clinical trial. JAMA Neurol..

[79] Rinaldi E., Puleo E., Consonni A., Miglietti M., Mantegazza R., Wang N., Peng S.L., Lewis K.E., Dillon S.R., Baggi F. (2025). Povetacicept (ALPN-303; TACI vTD-Fc), an enhanced, potent dual inhibitor of BAFF and APRIL, ameliorates experimental autoimmune myasthenia gravis in C57BL/6N mice. Front. Immunol..

[80] Brew S.G., Frey M., McCarthy D.P., Elhofy A., Nowak R.J. (2024). Antigen-specific immune therapy (CNP-106) for treatment of generalised myasthenia gravis: Rationale and design of first-in-human randomised controlled trial. BMJ Neurol. Open.

[81] Vu T., Durmus H., Rivner M., Shroff S., Ragole T., Myers B., Pasnoor M., Small G., Karam C., Vullaganti M. (2026). BCMA-directed mRNA CAR T cell therapy for myasthenia gravis: A randomized, double-blind, placebo-controlled phase 2b trial. Nat. Med..

[82] Zhang Y., Liu D., Zhang Z., Huang X., Cao J., Wang G., Li H., Li S., Zhang S., Zhang W. (2025). Anti-BCMA/CD19 CAR T cell therapy in patients with refractory generalized myasthenia gravis: A single-arm, phase 1 trial. eClinicalMedicine.

[83] Oh S., Mao X., Manfredo-Vieira S., Lee J., Patel D., Choi E.J., Alvarado A., Cottman-Thomas E., Maseda D., Tsao P.Y. (2023). Precision targeting of autoantigen-specific B cells in muscle-specific tyrosine kinase myasthenia gravis with chimeric autoantibody receptor T cells. Nat. Biotechnol..

[84] Oh S., Khani-Habibabadi F., O’Connor K.C., Payne A.S. (2025). Composition and function of AChR chimeric autoantibody receptor T cells for antigen-specific B cell depletion in myasthenia gravis. Sci. Adv..

[85] Oved K., Denkberg G., Reef S., Arman I., Pinzur L., Mazor R.D., Harel O., Erez S., Atiya Y., Dowery R. (2026). Preclinical characterization of MGAC-007, a first-in-class antigen drug conjugate for AChR^+^ myasthenia gravis (S14.010). Neurology.

